# Bio-inspired Hybrid Carbon Nanotube Muscles

**DOI:** 10.1038/srep26687

**Published:** 2016-05-25

**Authors:** Tae Hyeob Kim, Cheong Hoon Kwon, Changsun Lee, Jieun An, Tam Thi Thanh Phuong, Sun Hwa Park, Márcio D. Lima, Ray H. Baughman, Tong Mook Kang, Seon Jeong Kim

**Affiliations:** 1 Center for Self-powered Actuation, Department of Biomedical Engineering, Hanyang University, Seoul, 04763, Korea; 2 Department of Physiology, Samsung Biomedical Research Institute, Sungkyunkwan University School of Medicine, Suwon, 16419, Korea; 3 The Alan G. MacDiarmid NanoTech Institute, University of Texas at Dallas, Richardson, TX 75083, USA

## Abstract

There has been continuous progress in the development for biomedical engineering systems of hybrid muscle generated by combining skeletal muscle and artificial structure. The main factor affecting the actuation performance of hybrid muscle relies on the compatibility between living cells and their muscle scaffolds during cell culture. Here, we developed a hybrid muscle powered by C2C12 skeletal muscle cells based on the functionalized multi-walled carbon nanotubes (MWCNT) sheets coated with poly(3,4-ethylenedioxythiophene) (PEDOT) to achieve biomimetic actuation. This hydrophilic hybrid muscle is physically durable in solution and responds to electric field stimulation with flexible movement. Furthermore, the biomimetic actuation when controlled by electric field stimulation results in movement similar to that of the hornworm by patterned cell culture method. The contraction and relaxation behavior of the PEDOT/MWCNT-based hybrid muscle is similar to that of the single myotube movement, but has faster relaxation kinetics because of the shape-maintenance properties of the freestanding PEDOT/MWCNT sheets in solution. Our development provides the potential possibility for substantial innovation in the next generation of cell-based biohybrid microsystems.

Hybrid muscle systems, which include hybrid actuators composed of two-dimensional (2D) or three-dimensional (3D) structures, are generally produced by integrating living muscle cells and their scaffolds^1^,^2^,^3^. These hybrid muscles can be actuated by harmony of artificial structure and living entities, which allows their movement and interactions in a suitable environment, and they can efficiently act as a power source for micro- and nanosized biomedical devices^1^,^2^,^3^,^4^. The actuation, which is an essential function of the hybrid muscles, relies on the adhesiveness of the cells to the scaffold, organized scaffolds with flexibility and mechanical strength, and compatibility between the living cells and their scaffolds. Unlike general untransformable film type actuators^5^, flexible forms of 2D biohybrid actuators can be actuated with shape transformation such as bending, folding, and twisting. Therefore, flexible and biocompatible polymers such as polydimethylsiloxane^6^,^7^,^8^, poly-N-isopropylacrylamide^9^, polyaniline^10^ and poly(L-lactic acid)^11^ have long been favored as substrates for 2D muscle cell culture scaffolds^12^,^13^,^14^. Recently, instead of polymer-based scaffolds, various carbon-based 2D muscle scaffolds such as carbon nanotubes sheet^15^,^16^,^17^, graphene oxide film^18^, and graphene sheets^19^,^20^ have been reported to develop successful hybrid systems. These carbon-based scaffolds are attractive materials for constructing 2D cell-based biomedical applications^21^,^22^ due to their high electrical conductivities, high mechanical strengths, and biocompatibilities with cells^23^,^24^. Despite of the advanced progress on the fabrication of carbon materials, most of the carbon-based cell scaffolds still require complicated polymers and specific treatment protocols for stably attaching living cells^21^, and provides low actuation performance with inflexible property of the muscle scaffolds. In particular, the selection of an appropriate cell substrate is a principal factor in allowing stable and more extensive displacement of muscle scaffolds as a result of electrical stimuli^25^. One of the carbon-based cell culture substrates, a multi-walled carbon nanotubes (MWCNT) sheet, can effectively facilitate muscle movement by providing the structure needed for inducing self-alignment of myotubes on 2D muscle scaffolds^26^,^27^,^28^. Therefore, the main difference of graphene-based 2D surface (film or sheet) and MWCNT sheet is the possibility for inducing the self-alignment of myotubes on it. Furthermore, the MWCNT has a good cell-adhesion property due to its nano-fibrous structure. However, MWCNT sheet is severely compromised when placed in a liquid environment, making it extremely difficult to study in conjunction with cell culture media. Here, we introduce a new hybrid muscle composed of C2C12 skeletal muscle cells and the poly(3,4-ethylenedioxythiophene) (PEDOT)-coated MWCNT sheets that mimics the movement of the hornworm. This new PEDOT/MWCNT hybrid muscle has a hydrophilicity and biocompatibility^29^ that provides a cell-compatible environment and enhances its stability in cell culture medium. Moreover, the thickness and hydrophilicity of the PEDOT-coating is relatively easy to control by varying the concentration of 3,4-ethylenedioxythiophene during vapor phase polymerization (VPP) process. Additionally, the new hybrid muscles can potentially be applied to biomedical fields for use as a patch on an artificial organ or a biological sensor, because the cell-containing PEDOT/MWCNT sheets (10~20 nm) are easily modified to fit well on the substrate-outline as a functional thin nanomembrane. Moreover, compared with other hybrid culture systems based on carbide materials, the PEDOT/MWCNT hybrid muscle is fabricated by a simple process, providing the equivalent mechanical strength required for the endurance of the hybrid muscle system and is highly stable in solution during the whole period of culture. Furthermore, we successfully manufactured bio-inspired actuator capable of mimicking hornworm movement by using the patterned polytetrafluoroethylene (PTFE) solid mold. This patterning is very simple compared to the other previously reported systems^30^,^31^,^32^,^33^, because the PEDOT/MWCNT sheet has high shear stress that is enable to endure external pressure from PTFE solid mold. This innovative hybrid muscle could be widely used for diverse applications such as multifunctional actuators, integrated sensors, and biohybrid microsystems.

## Results

### PEDOT/MWCNT-based cell culture system

**Figure 1:**
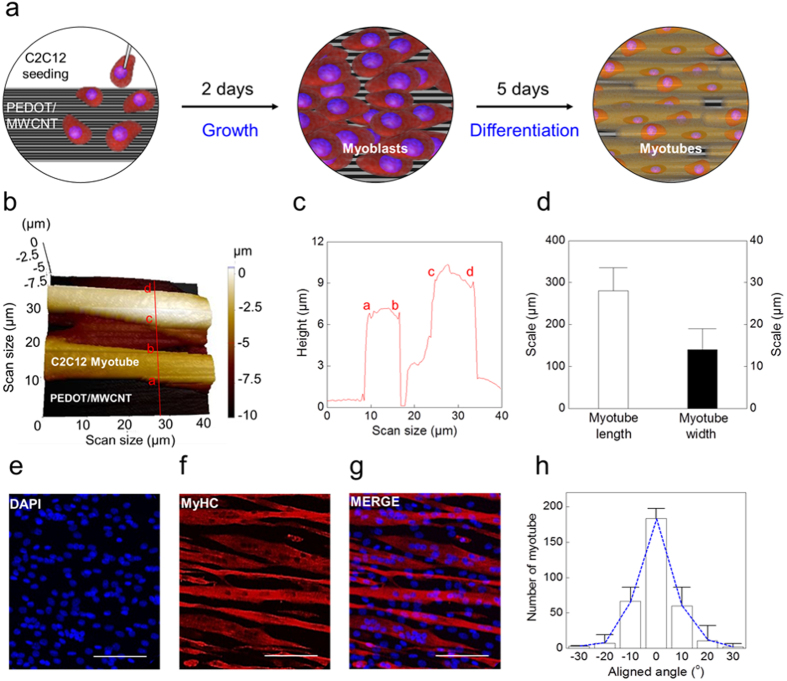
PEDOT/MWCNT-based cell culture platform and analysis of myotube alignment. (**a**) Schematic illustration of the cell culture protocol. (Left) C2C12 myoblasts seeded on a PEDOT/MWCNT sheet. (Middle) 2 days of cell culture. (Right) Aligned myotubes after 7 days of differentiation. (**b**) Scanning ion-conductance microscope (SICM) 3D image of the myotubes on the PEDOT/MWCNT sheet. The scan area is 40 × 40 μm. (**c**) Myotube width and height profiles were obtained from the cross-sectional line profile shown in (**b**). Each letter on the line profile corresponds to the same letter on the cross-section of (**b**). (**d**) The average myotube length and width were obtained from confocal microscope images of MyHC^+^ myotubes. (**e**–**g**) Confocal microscope images of MyHC^+^ myotubes grown on a PEDOT/MWCNT sheet. Scale bar = 50 μm. (**h**) Myotube alignment ratio analyzed by constructing an angular spread distribution histogram (n = 200). 93% of the myotubes were positioned in the range of −10° to +10°.

Figure 1a illustrates the culture process of C2C12 myoblasts on a PEDOT/MWCNT sheet. In contrast to the hydrophobic repulsion of a bare MWCNT sheet in solution (easily collapsed in the cell culture medium solution: Supplementary Fig. S1a,b)^34^, the hydrophilic surface of the PEDOT/MWCNT sheet can accept the seeded cells without any damage (Supplementary Fig. S1c,d). Furthermore, the PEDOT/MWCNT sheet has the mechanical strength (135 ± 8 MPa/(g/cm^3^)) compared to the bare MWCNT sheet (mechanical strength: 120 MPa/(g/cm^3^))^35^,^36^, and maintains its structure during cell culture. As shown in Supplementary Fig. S2, PEDOT polymer was uniformly coated on the MWCNT sheet surface (The average thickness of PEDOT/MWCNT sheet: 47 ± 12 nm).

As described in Methods, C2C12 myoblasts were differentiated for 7 days on the PEDOT/MWCNT sheet and the myotubes were characterized. The differentiated myotubes were arranged in parallel and well-aligned to the longer axis (longitudinal direction) of the PEDOT/MWCNT sheet (Fig. 1). Noninvasive imaging of the myotubes was performed using a scanning ion conductance microscope (SICM) to visualize the three-dimensional (3D) structure of the living cells. A SICM image of two parallel myotubes on the PEDOT/MWCNT sheet is shown in Fig. 1b. The vertical line profile over the SICM image shows that the myotubes are 10–15 μm in width and 6–10 μm in height (Fig. 1b,c). Collection of confocal microscope images of the myotubes revealed that the myotubes are 260 ± 30 μm in length, and 14 ± 10 μm in width (n = 100, Fig. 1d)^37^. This myotube size was consistent with other publications^32^. Differentiated myotubes were identified by immunostaining against a differentiation marker protein, myosin heavy chain (MyHC), and their alignment was analyzed (Fig. 1e–h). As shown in Fig. 1g, the myotubes were aligned in a parallel fashion along the longitudinal axis of the PEDOT/MWCNT sheet. To quantify the alignment ratio, a histogram of the myotube angular spread distribution histogram was constructed by analyzing the alignment angles of 380 cells with respect to the longitudinal direction of the PEDOT/MWCNT sheet (0°). Gaussian fitting of the histogram shows that all the cells are aligned within the range of −30° to +30°, with the highest alignment ratio at 0°(Ref. 38). In particular, 93% of the myotubes were aligned in the range of −10° to +10° (Fig. 1h). These results suggest that the longitudinally aligned surface structure of the PEDOT/MWCNT sheet determines and guides the axis of myotube alignment (Supplementary Figs S2 and S3). The differentiation rate and the alignment of the myotubes cultured on a bare MWCNT sheet were lower than those on the PEDOT/MWCNT sheet (Supplementary Fig. S4): the number of myotubes aligned in the same range (−10° to +10°) was 27% less than the myotube on PEDOT/MWCNT sheet (Supplementary Fig. S4d). Furthermore, the aligned myotubes grown on PEDOT/MWCNT sheet were well maintained up to 8 days of muscle differentiation (D8), as shown in Supplementary Fig. S5. The structure of the PEDOT/MWCNT sheet in the absence of C2C12 myoblasts was also visualized by SICM. The SICM image of the surface of the PEDOT/MWCNT showed a multi-parallel PEDOT ruffles several micrometers in width and ~100 nm in height (98 ± 11 nm). The longitudinal crests and grooves of the PEDOT ruffles were determined by the structure of the MWCNT bundles underneath the PEDOT membrane. As clearly shown in Fig. S3, the PEDOT/MWCNT sheet also has an alignment like bare MWCNT sheet. Therefore, the parallel ruffles running along the longitudinal axis of the PEPOT/MWCNT surface provide a structural foundation for the longitudinal alignment of differentiating myotubes.

### Single myotube actuation by electrical stimulation

**Figure 2:**
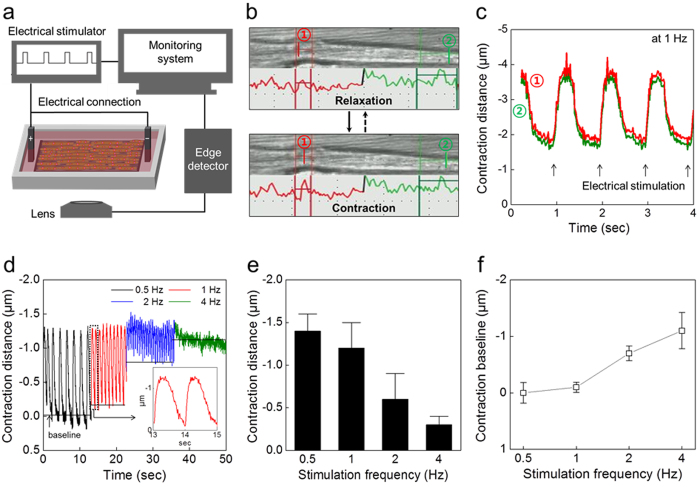
Single myotube contraction. (**a**) Schematic diagram of the device with an edge-detection system and electric field stimulation (EFS) for tracing contraction and relaxation. (**b**) Snapshot images from the monitoring system when a myotube relaxes and contracts. Red and green lines the indicate line scan profile of the left and right sides, respectively, of a single myotube. (**c**) Contraction distance changes of a single myotube at 1 Hz EFS. Red and green lines correspond to the movement of (1) and (2) indicated in (**b**). The average distance is 1.3 μm at 1 Hz EFS. (**d**) The frequency-contraction distance relationship in response to varying EFS frequencies. The inset shows the enlarged signal recorded at 1 Hz EFS. (**e**) Contraction distances were measured at each EFS frequency (1.4 ± 0.2 μm at 0.5 Hz; 1.2 ± 0.3 μm at 1 Hz; 0.6 ± 0.3 μm at 2 Hz; 0.3 ± 0.1 μm at 4 Hz; n = 500). (**f**) Baseline movement is increased as EFS frequency increases.

A single myotube cultured on the PEDOT/MWCNT sheet was contracted by electric field stimulation (EFS), and the contractile distance changes were measured by a microscopic edge detection system. A schematic diagram of the experimental setup is shown in Fig. 2a. EFS with a 10 ms step pulse duration (60–80 V) were applied at a variable frequency of stimulation (0.5–4 Hz). The distances of movement of two peak points (green and red) of the image line profile, corresponding to the right and left sides, respectively, of a single myotube, were traced to monitor the contraction and relaxation of a myotube (Fig. 2b). Real-time movement of the two points taken from a part of the myotube is displayed in detail in Supplementary Video S1. The myotube was actuated at 1 Hz and the corresponding contractile changes are displayed in Fig. 2c. The amplitude of the phasic contraction and relaxation distance of each point was ~1.6 μm with the similar kinetics. The actuation properties of the myotube with increasing frequency of EFS (0.5 to 4 Hz) are shown in Fig. 2d^32^. As the stimulation frequency increased, the contractile distance gradually decreased from 1.4 μm (at 0.5 Hz) to 0.3 μm (at 4 Hz) (Fig. 2e). At the same time, the contraction baseline moves up to 1 μm as the frequency increases from 0.5 to 4 Hz, suggesting that the tonic contraction of the muscle increases with higher frequency (Fig. 2f). The dependence of the contraction magnitudes and the baseline movement on increasing actuating EFS frequencies are mainly caused by the physiological temporal summation phenomena of the skeletal muscles^40^. At frequencies over 4 Hz, incomplete tetanus that was the result of partially relaxation from the previous contraction was observed because of muscle fatigue (green line in Fig. 2d).

### Fabrication of hornworm-like hybrid muscle

**Figure 3:**
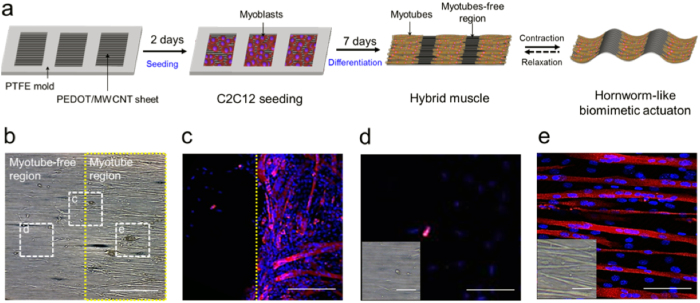
Fabrication of hornworm-like hybrid muscle. (**a**) Schematic illustration of the procedures for fabrication of the hybrid muscle using a PTFE mold. A window frame patterned PTFE mold was mounted on a PEDOT/MWCNT sheet and C2C12 cells were seeded in the open window regions of the mold. After 7 days of differentiation, the mold was removed and then the hornworm-like hybrid muscle was actuated by EFS. (**b**) A transmission electron microscope image shows myotube-free (left) and myotube regions (right). Confocal microscope images of the cells located in the dashed squares in (**b**) are presented in (**c**–**e**) (scale bar: 200 μm). (**c**) The PTFE mold separates the two regions and poorly-aligned myotubes are abundant at around the boundary region (yellow dashed line). (**d**,**e**) No myotube are observed in (**d**), but the well-aligned myotubes are plentiful in region (**e**). Transmission microscope images are inserted on the left of the confocal microscope images (Scale bar: 50 μm). Confocal image scale bars: 100 μm in (**c**); 25 μm in (**d**,**e**).

A custom-made solid mold in the shape of a window frame was fabricated from PTFE to separate the PEDOT/MWCNT sheet into two regions with and without proliferating myoblasts. The mold was mounted on the sheet and then cells were seeded on the exposed window surface of the sheet (Fig. 3a). After 7 days of differentiation, the mold was removed, and then the sheet covered with myotubes was detached carefully from the bottom of the slide glass. When the myotubes were fully differentiated on the PEDOT/MWCNT sheet, the intact sheet could be easily detached by removing the mold. The detached sheet remained flat and freestanding in the culture medium. As shown in the transmission and confocal microscope images of myotubes, cell migration was blocked by the mold, resulting in a myotube-free region (Fig. 3b–e). The boundary between the myotube and myotubes-free regions is indicated by the yellow line in Fig. 3c. In the myotube-free region, only a small number of nondifferentiating myoblasts were observed (Fig. 3d), but in the myotube region the differentiated myotubes were well-aligned (Fig. 3e). As a result, a hornworm-like biomimetic actuation platform powered by skeletal muscle cells was successfully fabricated using the hydrophilic PEDOT/MWCNT sheet.

### Contraction performances of hybrid muscle

**Figure 4:**
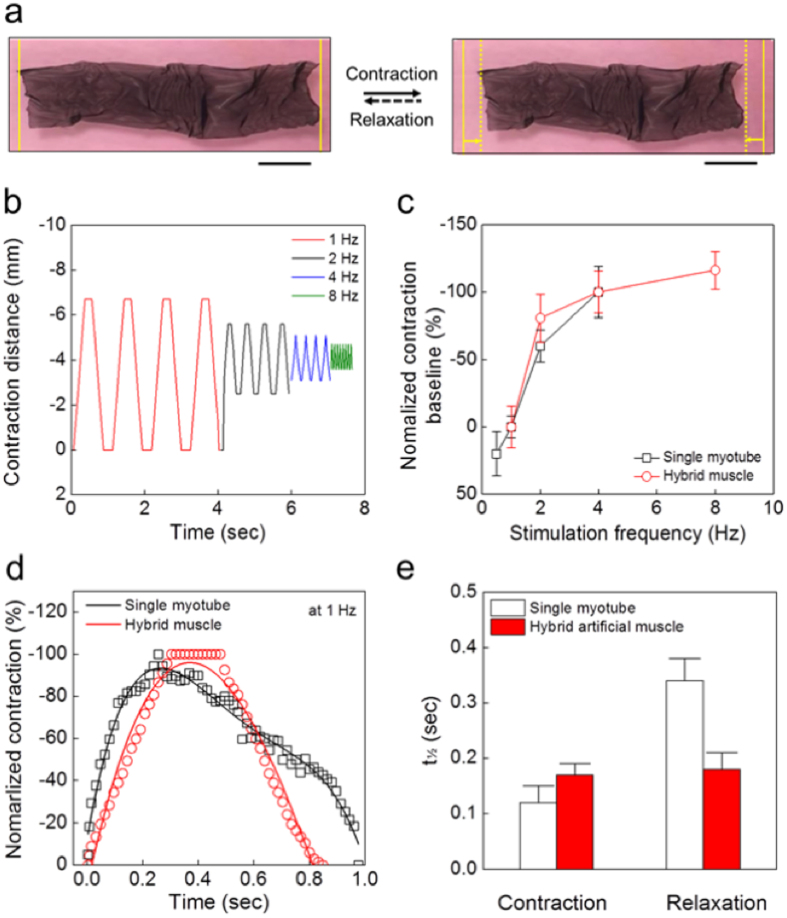
Contraction behaviors of hornworm-like hybrid muscle. (**a**) Photographs of the EFS-stimulated hybrid muscle (left, relaxation state; right, contraction state; scale bars, 10 mm). (**b**) Frequency-contraction distance relationship of the hybrid muscle in response to varying EFS frequencies (1–8 Hz). (**c**) Baseline movement in response to EFS frequency changes was compared between the hybrid muscle and the single myotube. Baseline movement is normalized to the amplitude at 1 Hz. (**d**) Normalized contraction behaviors of a single myotube and hybrid muscle system at 1 Hz. (**e**) The half-contraction and half-relaxation times (t_½_) were compared between the single myotube and the hybrid muscle system.

As illustrated in Fig. 3, we fabricated a hornworm-like hybrid muscle with a size of 60 mm (length) by 10 mm (width) (Fig. 4a). The horizontal axis of the actuator matched the longitudinal axis of the aligned myotubes. The hybrid muscle was placed in culture medium and stimulated to contract with varying frequencies of EFS. The horizontal movement of the freestanding actuator was captured on video and compared with the contractile behavior of a single myotube (Fig. 2 and Supplementary Video S2). The horizontal length changes in response to increasing EFS frequencies are shown in Fig. 4b. The contraction distances of the hybrid muscle decreased as the EFS frequency increased: ~0.7 mm and ~0.2 mm at 1 and 4 Hz, respectively (Fig. 4b). While the contraction baseline of a single myotube was slightly unstable over the course of stimulation (Fig. 2d), the contraction baseline of the hybrid muscle was stably maintained at a constant level during repetitive contraction and relaxation. This phenomenon was closely related to the recovery force of the hybrid muscle caused by the shape-maintenance property of the freestanding PEDOT/MWCNT sheet. As for single myotube contraction, the baseline length of the hybrid muscle shortened tonically as the EFS frequency increased (Fig. 4c). The characteristics of the baseline movement were qualitatively similar in both groups, suggesting that the movement was mainly determined by tonic contraction of the myotubes and was not hindered by the recovery force of the sheet.

The time courses of single twitches of the hybrid muscle were analyzed and compared with those of a single myotube (Fig. 4d). Contraction and relaxation of the actuator showed a symmetric behavior, with half contraction and half relaxation times (t_½_) of 0.17 ± 0.02 sec and 0.18 ± 0.03 sec, respectively. By contrast, a single twitch of the myotube was asymmetric, with half-contraction and half-relaxation time (t_½_) of 0.12 ± 0.03 sec and 0.34 ± 0.04 sec, respectively (n = 500) (Fig. 4e). As a result, it shows that the actuation behaviors of a hybrid muscle and a single myotube are clearly different. The asymmetric time course of myotube contraction and relaxation is considered to be a physiological phenomenon. During the excitation-contraction coupling processes of skeletal muscles, asymmetric time courses are normally observed for the electric action potential, the consequent intracellular calcium transient, and the contraction, with a slower decay time course during relaxation. The contraction time of the hybrid muscle was slightly longer than that of a single myotube, while the relaxation time was much shorter than that of a single myotube. This suggests that the shape-maintenance property of the PEDOT/MWCNT sheet does not hinder muscle contraction, but assists relaxation of the muscle by a recovery force originating from the sheet. In terms of energy saving, this recovery force generated by the sheet greatly reduces the energy consumption of the muscle as an actuating device. In addition, faster relaxation of the C2C12 muscle-powered biomimetic hybrid muscle permits the device to be actuated at a faster rate by reducing the refractory period of the muscle.

## Discussion

In this study we developed a new hybrid muscle based on a hydrophilic PEDOT/MWCNT sheet. This sheet provides a durable platform for muscle cell differentiation with a desired myotube alignment. The region covered with muscle cells acts as a power-generating component, and the muscle-free region enable to use for a flexible hinge or joint. For these reasons, this hornworm-like hybrid muscle can efficiently induce a horizontal length change in aqueous conditions. More importantly, our hybrid muscle has an enhanced shape-maintenance property generated by the PEDOT/MWCNT sheet in solution that greatly reduces the inner energy consumption and external energy requirement for actuation. Development of this technique is a substantially innovation in the generation of multifunctional actuators or sensors based on carbides and nanomaterials. The biocompatible properties of our device mean that it can be used as a culture platform for a variety of cells, including muscle cells (skeletal, cardiac, smooth), neurons, endocrine cells, and vascular networks. The higher electrical conductivity of the PEDOT/MWCNT platform allows bidirectional electrical communications between the attached cells (muscle and nerves) and the platform, with the maintenance of functional integrity. The advantages of PEDOT/MWCNT mean that it is applicable to many systems by modification of the device into different shapes, such as yarns, coils, and more complicated 3D structures. It provides great insights for the generation of innovative multifunctional hybrid muscle or sensors that can be applied in biomedical systems.

## Materials and Methods

### Materials

Multi-walled carbon nanotube (MWCNT) sheets were drawn from ~400 μm high MWCNT forests^27^ that were grown by chemical vapor deposition on iron catalyst-coated silicon wafers using acetylene (C2H2) gas as the carbon precursor^36^. Iron(III)p-toluene sulfonate hexahydrate (Fe(III)PTS; Mw, 677.52), pyridine (anhydrous, 99.8%), 1-butanol (≥99%), and 3,4-ethylenedioxythiophene (EDOT) monomer (97%) were purchased from Sigma-Aldrich. Sulfuric acid solutions (2N; 1 M H_2_SO_4_) were purchased from Daejung Chemicals.

### Fabrication of PEDOT/MWCNT sheets

A three-layer of MWCNT sheet (10 mm wide, and 60 mm long) was prepared on a slide glass. For the polymerization of poly(3,4-ethylenedioxythiophene) (PEDOT), a 20 wt% solution of Fe(III)PTS in butanol (with 1.6 vol% of subsequently added pyridine) was used. The Fe(III) acted as the oxidizing agent and PTS (or tosylate) acted as the anionic dopant. Fe(III)PTS-pyridine-butanol solutions containing 8 wt% of oxidant were made by diluting this stock solution with butanol. 8 wt% of oxidant solution was dropped over a carbon nanotube aerogel sheet stack, thereby delivering 100 μl of diluted solution to the carbon nanotube sheet stack. The carbon nanotube sheet stack was then dried at 60 °C for 2 min to evaporate the solvent. Densified Fe(III)PTS-containing sheets were thereby obtained. Vapor phase polymerization (VPP) to coat the MWCNTs with PEDOT was accomplished by exposing these Fe(III)PTS-containing sheets to EDOT vapor in a VPP chamber for 1 h at 60 °C. After VPP, the PEDOT-containing MWCNT sheets were washed three times using ethanol.

### Myoblast culture and differentiation on PEDOT/MWCNT sheets

Methods for C2C12 myoblast culture, differentiation, and immunostaining were described in our previous study^41^. In brief, C2C12 myoblasts were cultured in Dulbecco’s modified Eagle’s medium (DMEM, Welgen) supplemented with 15% fetal bovine serum (FBS, GIBCO/BRL). The cultured myoblasts (<80% confluent) were detached by 0.5% trypsin-ethylenediaminetetraacetic acid treatment to prepare the cell suspension. A PEDOT/MWCNT sheet was prepared on a slide glass, and then placed in a culture dish. A window frame shaped polytetrafluoroethylene (PTFE) plate mold (25 mm wide, and 60 mm long) was prepared and mounted on the PEDOT/MWCNT sheet. The mold had a series of open square window regions (10 mm width and length) at 5 mm intervals along the longitudinal direction of the mold. Thus, this patterned structure of the mold gives a hornworm-like cell culture platform. The prepared C2C12 cell suspension was uniformly seeded within the window regions of the mold at a cell density of 30,000/cm^2^. When required, the cells were seeded and cultured in the absence of the mounted PTFE mold. After the cells had settled down on the sheet, the entire culture dish was carefully filled with a volume of growth medium sufficient to cover the cells on the slide glass. After 2 days of cell growth, myoblasts (>95% confluent) were induced to differentiate into myotubes by replacing the growth medium with differentiation medium (DMEM supplemented with 2% horse serum, GIBCO/BRL) for the next 7 days, without applying external electrical stimulation or mechanical stretching.

### Immunocytochemistry and alignment assay

Differentiated myotubes on PEDOT/MWCNT sheets were fixed with 4% cold paraformaldehyde (Santa Cruz, 15 min), and permeabilized with 0.5% Triton-X (15 min), and the sample was blocked with 5% horse serum solution (30 min). Fixed samples were incubated with anti-myosin heavy chain (anti-MyHC) antibody at 4 °C overnight, and then incubated with Alexa-flour 647 nm anti-mouse antibody (Invitrogen) at room temperature for 2 hrs. For 2-(4-amiddinophenyl)-1H-indole-6-carboxamidine (DAPI) staining of the cell nucleus, the sample was put onto the DAPI antibody (1:10000, Sigma). Finally, the samples were mounted with prolong-mounting solution (Thermoscientific). Multi-nucleated MyHC-positive (MyHC^+^) myotubes were imaged with a confocal microscope (LSM 710, Zeiss) at the Research Core Facility, SBRI. The orientation of the myotubes was quantified by measuring the angle between the longitudinal axis of the cells and the direction of alignment of the PEDOT/MWCNT sheet. The myotube alignment ratio was analyzed by constructing a histogram of the myotube angular spread distribution^42^.

### Scanning ion-conductance microscopy (SICM)

Surface structures of the differentiated myotubes cultured on the PEDOT/MWCNT sheet were obtained using the SICM nano-imaging technique^43^. SICM images were obtained with the XE-Bio System (Park Systems). Myotube samples were embedded in phosphate-buffered saline (PBS) solution and placed on an x-y flat scanner stage (XE-Bio System) mounted on an inverted microscope (IX71, Olympus). The SICM probe glass nano-pipette (inner diameter of ~100 nm) was fabricated from borosilicate glass tubing (Warner Instruments) using a CO_2_-laser-based micropipette puller (P-2000, Sutter Instruments, CA) and filled with PBS electrolyte solution. Using the approach-retract scanning (ARS) or hopping modes of SICM imaging^43^, 3D cell surface images of the live myotubes were successfully obtained in a non-invasive manner.

### Microscopic single-cell contraction measurement

Differentiated myotubes on a PEDOT/MWCNT sheet were mounted on an inverted microscope (IX71, Olympus) and perfused with DMEM culture medium. Single myotube contraction was elicited by EFS using 10 ms voltage pulses (60–80 V) at varying the frequencies (0.5–4 Hz). Then, cell shortening distances, rates of shortening and relaxation were recorded online using a video-edge detection system in bright field (IonOptix, Milton, MA, USA) at acquisition frequency of 250 Hz, and were analyzed by IonWizard v6.3 software (IonOptix)^44^.

### Analysis of hybrid muscle

The hybrid muscle was placed in DMEM culture medium and actuated by applying EFS at varying frequencies (1–8 Hz). The contraction changes of the hornworm-like biomimetic actuator in the longitudinal direction were recorded with a custom-made video camera. The recorded image was captured at 40 frames/sec using ImageJ image processing software (http://rsb.info.nih.gov/ij/), and analyzed to estimate the performance of the actuator. The phasic contraction distance and baseline movement of the freestanding actuator in response to the stimulation frequency were quantified.

## Additional Information

**How to cite this article**: Kim, T. H. *et al*. Bio-inspired Hybrid Carbon Nanotube Muscles. *Sci. Rep.* **6**, 26687; doi: 10.1038/srep26687 (2016).

## Supplementary Material

Supplementary Information

Supplementary Video 1

Supplementary Video 2
