# Supplementary material for: Bio-inspired Hybrid Carbon Nanotube Muscles
Source: Sci Rep. 2016 May 25;6:26687. doi: 10.1038/srep26687 (PMC4879622; doi:10.1038/srep26687)
Supplement: Supplementary Information [file srep26687-s1.pdf]

Supporting Information

Bio-inspired Hybrid Carbon Nanotube Muscles

Tae Hyeob Kim[1,*], Cheong Hoon Kwon[1,*], Changsun Lee[1], Jieun An[2], Tam Thi Thanh Phuong[2], Sun Hwa Park[2], Márcio D. Lima[3], Ray H. Baughman[3], Tong Mook Kang[2,†], and Seon Jeong Kim[1,†]

[1]Center for Self-powered Actuation, Department of Biomedical Engineering, Hanyang University, Seoul, 04763, Korea.
[2]Department of Physiology, Samsung Biomedical Research Institute, Sungkyunkwan University School of Medicine, Suwon, 16419, Korea
[3]The Alan G. MacDiarmid NanoTech Institute, University of Texas at Dallas, Richardson, TX 75083, USA

***** These authors contributed equally to this work.
[†]To whom correspondence should be addressed: sjk@hanyang.ac.kr and tongmkang@skku.edu

Supplementary Movies

Movie S1. Real-time tracing of the contraction and relaxation of a single myotube.

Movie S2. Contraction of hornworm-like hybrid muscle.

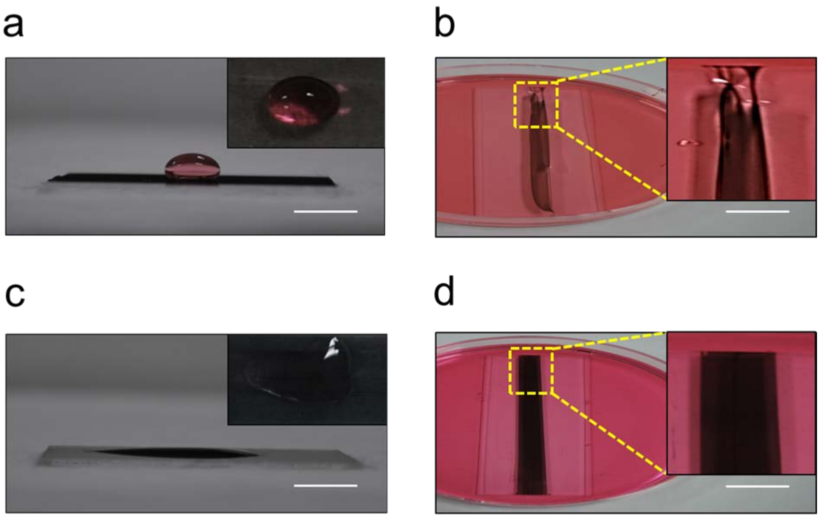

Figure S1. Comparison of the hydrophilicity of a bare MWCNT and a PEDOT-coated MWCNT sheets. (a) When cell culture medium (DMEM) is dropped on a bare MWCNT, the drop on it maintains a higher contact angle (Scale bar: 1 cm). (b) Fully-sunk bare MWCNT sheet with partially damaged structure in culture medium (Scale bar: 2 cm). (c) While, the drops on a PEDOT/MWCNT sheet, spread widely, indicating its hydrophilicity (Scale bar: 1 cm). (d) Fully-sunk PEDOT/MWCNT sheet showing the shape-maintenance property in culture medium (Scale bar: 2 cm)

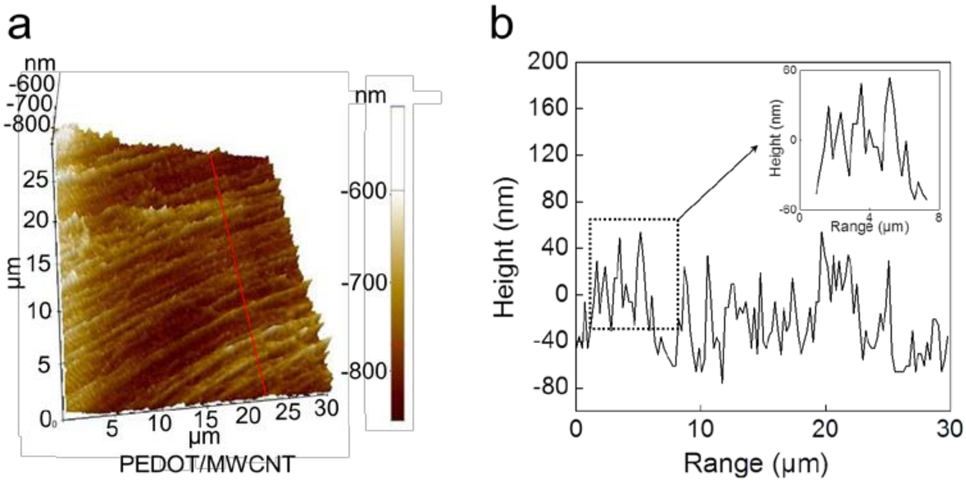

Figure S2. SICM image of the PEDOT/MWCNT sheet surface. The manufactured PEDOT/MWCNT sheet is prepared on a slide glass and placed in PBS buffer solution, and then a SICM image of the surface is obtained. (**a**) A three-dimensional SICM image of a PEDOT/MWCNT sheet surface. Scan size: 30 × 30 μm. (**b**) Graph of the SICM image. The inset shows an enlarged line profile from the dashed box.

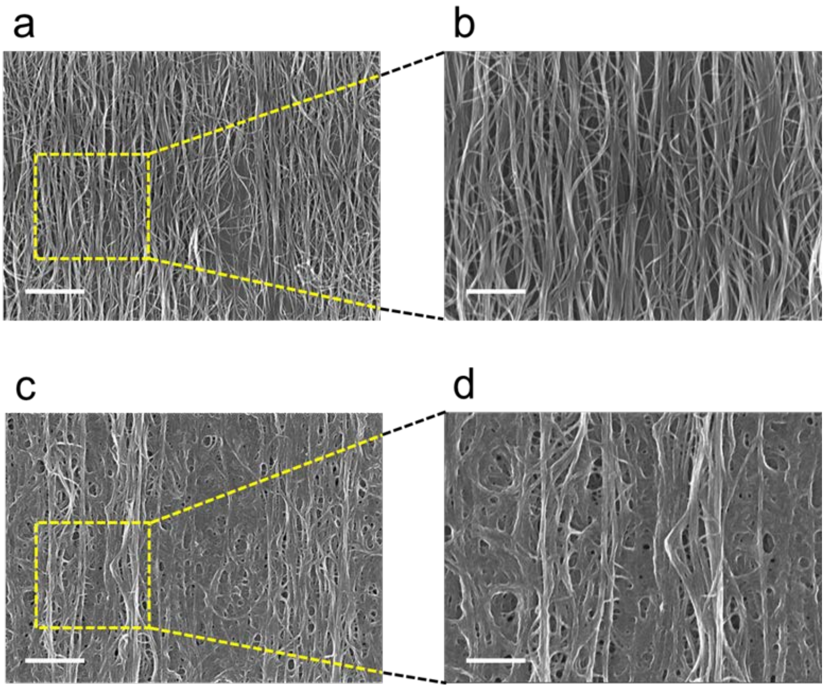

Figure S3. Comparison of each surface of a bare MWCNT sheet and a PEDOT-coated MWCNT sheet. (**a**) The surface image of a bare MWCNT sheet. Each carbon nanotube is well-aligned with an axial direction (Scale bar: 1 μm) and (**b**) is a close-up SEM image of Fig. S3a (Scale bar: 250 nm). (**c**) The surface image of a PEDOT-coated MWCNT sheet. Compared to a bare MWCNT sheet, CNT bundles are densely placed with coating PEDOT polymer, and still kept the alignment from the bare MWCNT sheet (Scale bar: 1 μm). (**d**) a close-up SEM image of Fig. S3c (Scale bar: 250 nm).

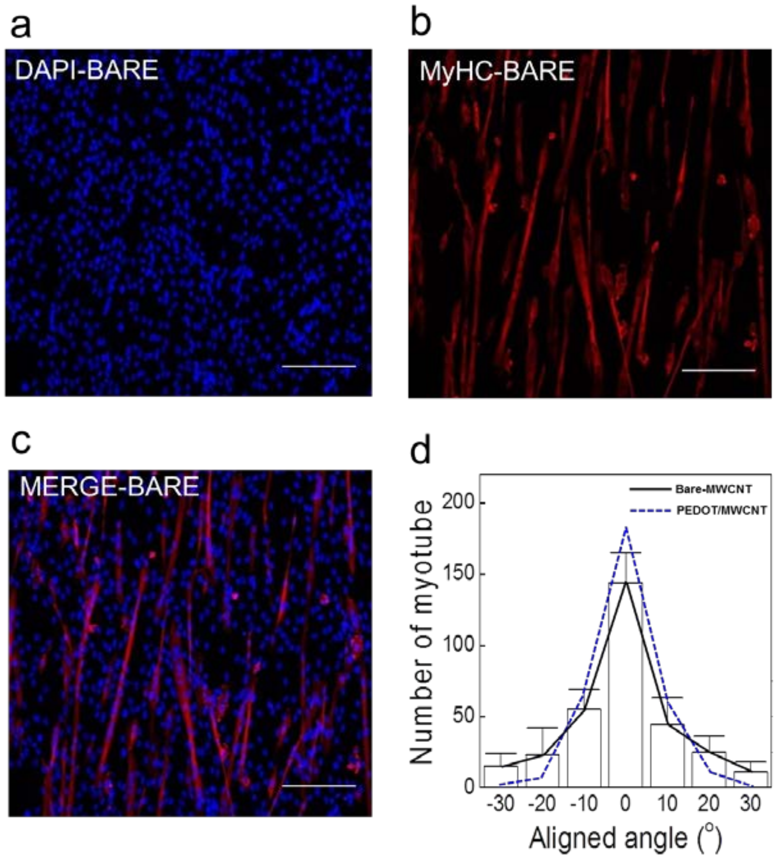

Figure S4. MyHC⁺ C2C12 myotubes differentiated on bare MWCNT sheet. The degree of differentiation and cell alignment on the bare MWCNT sheet is lower than that on the PEDOT/MWCNT sheet. (**a**) Nuclear staining with DAPI. (**b**) MyHC immunocytochemistry of the myotubes. (**c**) Merged image of DAPI and MyHC (Scale bars: 100 µm). (**d**) Myotube alignment ratio analyzed by constructed angular spread distribution histogram. About 46 % of the myotubes are aligned with the direction of alignment of the bare MWCNT sheet (0° aligned angle, n = 70).

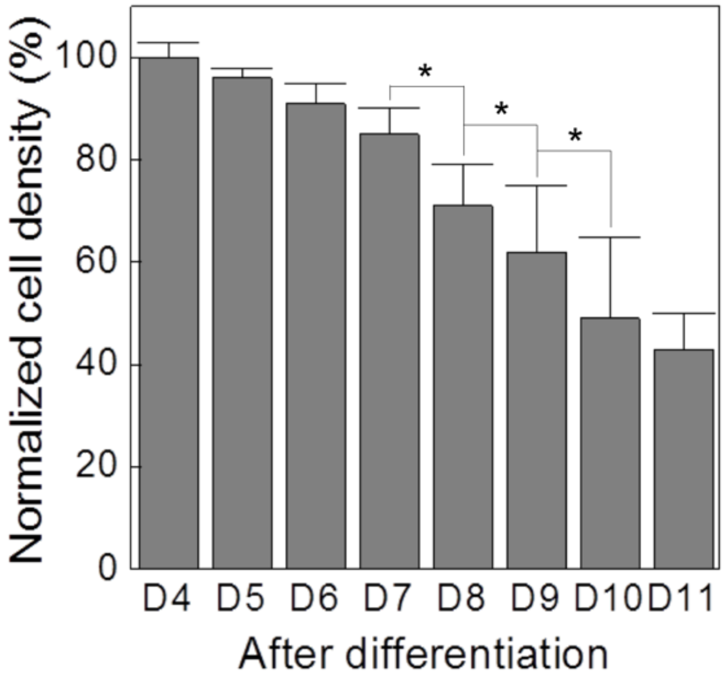

Figure S5. The plot of the normalized cell densities according to the time (after differentiation) (* $p<0.05$, $n=5$).
